# A Novel End-To-End Feature Selection and Diagnosis Method for Rotating Machinery

**DOI:** 10.3390/s21062056

**Published:** 2021-03-15

**Authors:** Gang Wang, Yang Zhao, Jiasi Zhang, Yongjie Ning

**Affiliations:** 1 The National Joint Engineering Laboratory of Internet Applied Technology of Mines, China University of Mining and Technology, Xuzhou 221000, China; wanggang@cumt.edu.cn; 2 School of Information and Control Engineering, China University of Mining and Technology, Xuzhou 221000, China; TS20060228P31@cumt.edu.cn (J.Z.); TS16060245P3@cumt.edu.cn (Y.N.); 3 State Grid Puyang Power Supply Company, Puyang 457000, China

**Keywords:** MIVs, WBDA, feature selection, rotating machinery, noise diagnosis

## Abstract

Feature selection is to obtain effective features from data, also known as feature engineering. Traditional feature selection and predictive model learning are separated, and there is a problem of inconsistency of criteria. This paper presents an end-to-end feature selection and diagnosis method that organically unifies feature expression learning and machine prediction learning into one model. The algorithm first combines the prediction model to calculate the mean impact value (MIVs) of the feature and realizes primary feature selection for the prediction model by selecting the feature with a larger MIV. In order to take into account the performance of the feature itself, the within-class and between-class discriminant analysis (WBDA) method is proposed, and combined with the feature diversity strategy, the feature-oriented secondary selection is realized. Eventually, feature vectors obtained by two selections are classified using a multi-class support vector machine (SVM). Compared with the modified network variable selection algorithm (MIVs), the principal component analysis dimensionality reduction algorithm (PCA), variable selection based on compensative distance evaluation technology (CDET), and other algorithms, the proposed method MIVs-WBDA exhibits excellent classification accuracy owing to the fusion of feature selection and predictive model learning. According to the results of classification accuracy testing after dimensionality reduction on rotating machinery status, the MIVs-WBDA method has a 3% classification accuracy improvement under the low-dimensional feature set. The typical running time of this classification learning algorithm is less than 10 s, while using deep learning, its running time will be more than a few hours.

## 1. Introduction

Bearings are easily damaged parts in rotating machinery, and approximately 50% of motor faults are bearing related [1,2]. The machinery running noise is a type of mechanical wave, which includes a wealth of information about machine status, and propagates energy to the surrounding environment through vibration [3,4]. Both noise and vibration are caused by the elastic deformations of the rotor, and therefore, the machinery running noise is a good indicator as the vibration signal [3,5]. Compared with vibration diagnostics, the noise diagnostics have the characteristics of non-contact measurements, convenient sensor installation, no influence on machinery operation, and online monitoring. Noise diagnostics are especially suitable for occasions where the vibration signal is not easy to measure [4]. This paper studies the rotating machinery fault diagnosis method based on noise signals.

Rotating machinery noise diagnosis achieves diagnosis of machinery working conditions by monitoring the elastic waves induced by deformations, exfoliations, or cracks. Fault diagnosis can be regarded as a pattern recognition problem; artificial intelligence (AI) has attracted great attention and shows promise in rotating machinery fault recognition applications [6]. The rotating machinery fault diagnosis based on AI includes sensing, data acquisition, feature extraction, dimensionality reduction, and fault classification. Among them, feature extraction and dimension reduction are the most critical steps in the workflow [7]. They are related to the upper limit of the fault identification accuracy of the subsequent classification algorithm. Too much redundant information in high-dimension feature vectors may lead to curse of dimensionality and increasing calculation time. The principle of selection is to try not to miss a feature that may be useful, but not to abuse too many features. To extract the features, many signal processing methods have been used in the area of rotating machine health monitoring and diagnosis, such as time-domain and frequency-domain feature parameters processing [8,9,10], discrete wavelet transform (DWT) [11], empirical mode decomposition (EMD) [12], time-frequency analysis (TFA) [13], Mel-frequency cepstrum (MFC) [14], and Shannon entropy [15]. Among them, Shannon entropy features have been widely used in machine health monitoring recently. For example, the instantaneous energy distribution-permutation entropy (IED-PE) [16], the improved multiscale dispersion entropy (IMDE) [17], the composite multi-scale weighted permutation entropy (CMWPE) [18], the stationary wavelet packet Fourier entropy (SWPFE) [19], and similarity-fuzzy entropy [20] have been proposed to construct the sensitive feature for rolling balling heath monitoring. However, the construction of good sensitive features requires manual experience, which is called feature engineering problem. With the application of deep learning, some feature self-encoding methods are adopted [21]. However, the difficulty of deep learning is how to evaluate the contribution of representation learning to the final system output. At present, a more effective method is to use the final output layer as predictive learning and other layers as representation learning.

Feature selection is to select an effective subset of the original feature set, so that the model trained based on this feature subset has the highest accuracy. A direct feature selection algorithm is a subset search algorithm, and a commonly used method is to adopt a greedy strategy, such as forward search or reverse search. Subset search algorithms are divided into two types: filter and wrapper. The filter method is a feature selection method that does not depend on a specific machine learning model, while the wrapper method is a method that uses the accuracy of subsequent machine learning models as a feature selection criterion. Another feature learning is feature extraction, which is to project the original feature in a new space to obtain a new feature representation, such as principal component analysis (PCA) and auto-encoder. In existing feature selection or feature extraction algorithms, PCA transforms the original data into linearity-independent data via linear transformation, and it can be used to extract the main feature components of the data [22]. PCA expands features in the direction in which the covariance is the largest so that the obtained low dimensional features have no corresponding physical meaning. Chen B. et al. achieved selection and dimensionality reduction of intrinsic mode function (IMF) components of motor bearing via distance evaluation technology (DET) and utilized dimensionality-reduced feature vectors as input vectors for a support vector machine (SVM) [23]. Lei et al. proposed compensative distance evaluation technology (CDET) with enhanced dimensionality reduction performance, and they applied this to feature dimensionality reduction of bearing vibration signals [24]. CDET selects the features that have the smallest distance within the cluster and the largest distance between clusters. PCA, DET, and CDET do not consider the characteristics of the classification network. Melih Kuncan et al. proposed a feature extraction method based on one-dimensional ternary patterns (1D-TP) obtained from comparisons between neighbors of each value on vibration signals for bearing fault classification [25]. To solve the problems of variable redundancy and model complexity in the prediction model, Xu et al. combined the neural network and the mean impact value (MIV) for wind power prediction [26]. In addition, methods based on decision trees or GBDT for feature extraction or dimensionality reduction have been used in machinery diagnostics. Madhusudana et al. used the decision tree technique to select prominent features out of all extracted features [27]. Li et al. proposed a wrapped feature selection algorithm based on XGBoost which used the importance measure of XGBoost as a feature subset search heuristic, and it was verified on 8 data sets [28]. Aiming at the problem of variable working conditions of rotating equipment, Wu et al. proposed a deep autoencoder feature learning method and applied it to fault diagnosis of rotating equipment [29].

In terms of feature classification, neural networks [30,31] and SVM [32,33] have been widely applied in machinery diagnosis. Han et al. compared the performance of random forest, artificial neural networks and SVM methods in the intelligent diagnosis of rotating equipment [34]. Hu et al. utilized the wavelet package transform and SVM ensemble technology for fault diagnosis [35]. Liu et al. proposed a genetic algorithm (GA) based self-adaptive resonance demodulation technique [36]. Zhu et al. proposed a fault diagnosis method based on an SVM optimized by the GA [37]. Han et al. combined EMD, particle a swarm optimization SVM (PSO-SVM), and fractal box dimensions for gear fault feature extraction and fault classification [38]. Indeed, heuristic searching methods, such as the GA and simulated annealing [39] and tabu searching methods [40] have also been applied in feature classification. In addition, ensemble learning and deep neural networks are widely used in fault diagnosis [41]. Zhou et al. proposed a novel bearing diagnosis method based on ensembled empirical mode decomposition (EEMD) and weighted PE and further enhanced the classification accuracy by a mixed voting strategy and a similarity criterion [42]. Aiming at the problem of big data analysis, Wu et al. proposed a two-stage big data analytics framework and achieved a high-level of classification accuracy [43].

The conventional rotating machinery diagnosis algorithms separate the complementarity of the feature selection algorithm and classification network in feature selections. To this end, this paper proposes an end-to-end feature selection and diagnosis method that organically unifies feature expression learning and machine prediction learning into one model. This method realizes the compromise between the two types of algorithms and applies it to the state classification of machinery. First, based on the modified MIVs algorithm, our algorithm not only achieves feature selection for noise signals based on the contributions of independent variables to classified networks but also solves the randomness problem of MIVs value. By eliminating the features that have less influence on the classification, this step realizes the primary feature selection oriented to the classification network. Second, in order to characterize the metric ability of the feature itself, a new between-class sorting WBDA algorithm was introduced into the intra-class and inter-class aggregation degree calculation, and feature diversity selection strategy is proposed to prevent the phenomenon that the calculated WBDA of the features in the same category are relatively large. Experimental results show that this feature diversity selection strategy can effectively improve the accuracy of the algorithm. Thus, secondary selection of features was achieved through feature indexability. Since there are few faulty data in industrial applications, it is hoped that the diagnosis algorithm can run online. The classification network uses the SVM to compute the actual classification accuracy and removes the local optimal solution through the Monte Carlo method. The present paper compares the proposed algorithm with the MIV algorithm for network variable selection, the CDET algorithm based on variable selection, and the variable dimensionality reduction algorithm PCA. After selecting features with the same dimensions, the proposed algorithm is found to have better classification accuracy than the other methods, which verifies its superiority.

This paper is organized as follows. Section 1 introduces the background, motivation, and a brief literature review of Feature learning and feature classification. Section 2 constructs the machinery noise feature set which is used for testing in Section 6. In Section 3, a bearing noise diagnosis algorithm based on network variable selection and WBDA, named MIVs-WBDA, is proposed. Since feature classifications were achieved by an SVM, Section 4 introduces two classifier parameter optimization algorithms for the SVM: PSO algorithm and the GA. Section 5 summarizes the procedures of the MIVs-WBDA. Section 6 describes the simulation testing. Finally, Section 7 presents our conclusions and some further remarks.

## 2. Feature Extraction

**Figure 1:**
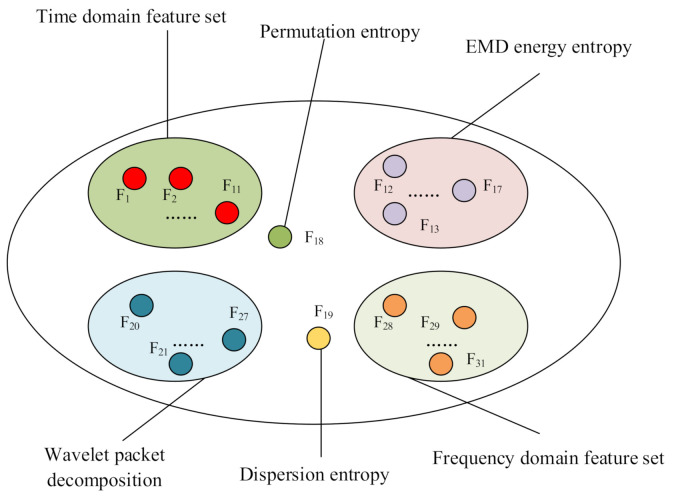
Feature pool.

In practical applications, it is difficult to determine which features are key ones in advance, and classifiers based on different features may have significantly different performance. For the application of this paper, in order to verify whether the proposed feature selection algorithm can select the most suitable features from the undetermined feature set, a large number of features used in the previous literatures are constructed as the candidate feature set. These features form a feature pool. As a test, a total of 31 features were constructed in this article, which were divided into 6 classes, as shown in Figure 1.

### 2.1. Tranditional Time Domain Feature Set

Traditional time domain and statistic features are a powerful tool which can characterize the change of bearing vibration signals when faults occur [44]. The time-domain characteristics are more significant, which can be directly obtained from the monitoring signal, and reflect the change of energy amplitude on the time scale of the signal. It is a common index that can be used for rapid diagnosis. This paper uses 11 features shown in Table 1. Herein, $x_{i}$ refers to the *i*-th measurement of the time domain signal, $s_{i}$ refers to the *i*-th frequency domain value based on the short-time Fourier transform (STFT), and $x_{i}^{’}$ refers to the i-th of $x_{i}$ in ascending order, where *N* is an even number. Subscript *i* takes values from 1 to *N*. $F_{j}\left(j=1\ldots 11\right)$ refers to the *j*-th feature of the signal. μ is the mean of signal x, and σ is the variance. These features are calculated for every short-time frame of bearing noise signal.

### 2.2. Empirical Mode Decomposition Energy Entropy

Features 12 to 17 are empirical mode decomposition energy entropy. EMD is a signal analysis method proposed by Dr. Huang in 1998 [45]. It is an adaptive data processing or mining method, which is very suitable for the processing of nonlinear and non-stationary time series. The EMD extraction method is given as the following:

(a) Decompose bearing noise signals into some IMFs.

(b) Calculate the energy of all IMFs

(1) $$Energy\_imf\left(j\right)=\overset{N}{\underset{i=1}{\sum }}{\left|imf\left(i,j\right)\right|}^{2}\;j=1\cdots M$$

(c) Calculate the energy entropy of all IMFs

(2) $$IMF\_entropy\left(j\right)=-\overset{N}{\underset{i=1}{\sum }}\frac{{\left|imf\left(i,j\right)\right|}^{2}}{Energy\_imf\left(j\right)}log\frac{{\left|imf\left(i,j\right)\right|}^{2}}{Energy\_imf\left(j\right)}\;j=1\cdots M$$

(d) Calculate the energy entropy of the whole original signal

(3) $$Energy\_total=-\overset{M}{\underset{j=1}{\sum }}Energy\_imf\left(j\right)$$

(4) $$EMD\_entropy=-\overset{N}{\underset{i=1}{\sum }}\frac{{\left|Energy\_imf\left(j\right)\right|}^{2}}{Energy\_total}log\frac{{\left|Energy\_imf\left(j\right)\right|}^{2}}{Energy\_total}$$

(e) Construct the feature vector with the first six IMF_entropy(j) and EMD_entropy

(5) $$\left[F_{12},F_{13},\cdots ,F_{17}]=[EMD\_entropy,IMF\_entropy\left(1\right),\cdots ,IMF\_entropy\left(5\right)\right]$$

**Figure 2:**
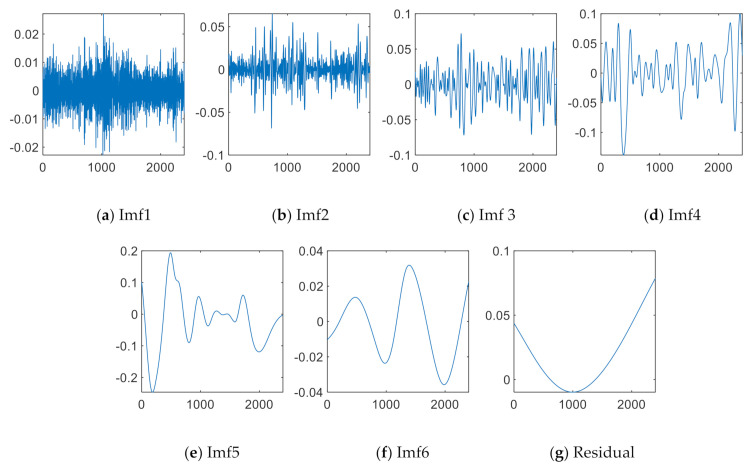
Empirical mode decomposition (EMD) of a sample.

Figure 2 shows the empirical mode decomposition diagram of a sample.

### 2.3. Permutation Entropy

Feature 18 is permutation entropy. Permutation entropy algorithm is a kind of vibration mutation detection method, which can conveniently locate the mutation time of the system and has the ability to detect the small change of the signal.

The calculation steps of PE are as follows:

(1) Let the length of time series *x*_j_ (*j* = 1, 2, ..., *N*) be *N*, and define an embedding dimension *m* and a time delay *D*.

(2) The signal is reconstructed in phase-space to obtain *k* (*k* = *N*− (*m* − 1)*d*) reconstructed components, and each component is represented by *X*_i_ = {*x*(*i*),*x*(*i* + *d*),…, *x*(*i* + (*m* − 1)*d*}.

(6) $$X=\left[\begin{array}{cccc} x\left(1\right) & x\left(1+d\right) & \cdots & x\left(1+\left(m-1\right)d\right)\\ \vdots & & \cdots & \vdots \\ x\left(j\right) & x\left(j+d\right) & \cdots & x\left(j+\left(m-1\right)d\right)\\ \vdots & & \cdots & \vdots \\ x\left(k\right) & x\left(k+d\right) & \cdots & x\left(N\right) \end{array}\right]$$

(3) The inner part of each subsequence ***X****_i_* is sorted incrementally, that is, $x\left(i+\left(j_{1}-1\right)d\right)\leq x\left(i+\left(j_{2}-1\right)d\right)\leq \ldots \leq x\left(i+\left(j_{m}-1\right)d\right)$. When sorting, if two values are equal, they are sorted according to the subscript n of $j_{n}$. In this way, an ***X****_i_* is mapped to a sequential pattern $\pi_{j}=\left(j_{1},j_{2}\ldots j_{m}\right)$, which is one of all possible sequential patterns of *m* number. Therefore, every m-dimensional subsequence *X_i_* is mapped to one of *m*! permutations.

(4) Calculate the times of each permutation pattern $\pi_{j}$ appearing in *m*! permutations, denoted as $f\left(\pi_{j}\right)$, then the probability of each permutation pattern appearing is defined as

(7) $$P\left(\pi_{j}\right)=\frac{f\left(\pi_{j}\right)}{\sum_{j=1}^{m!}f\left(\pi_{j}\right)}$$

(5) The permutation entropy of time order is defined as

(8) $$H_{p}\left(m\right)=-\overset{m!}{\underset{j=1}{\sum }}P\left(\pi_{j}\right)logP\left(\pi_{j}\right)$$

Obviously, $0\leq H_{p}\left(m\right)\leq \log(m!)$. In general, $H_{P}\left(m\right)$ is normalized to 0–1, and $H_{P}=\frac{H_{P}\left(m\right)}{\log\left(m!\right)}$ is defined for this purpose.

### 2.4. Dispersion Entropy

Features 19 is dispersion entropy. Rostaghi [46] et al. gave the detailed calculation steps of DE as follows. For a given univariate signal of length *N*: $x=\left\{x_{1},x_{2},\ldots ,x_{N}\right\}$, the DE algorithm includes 4 main steps:

(1) First, $x_{j},\left(j=1,\;2,\cdots ,N\right)$ are mapped to *c* classes, labeled from 1 to *c*. To do so, there are a number of linear and nonlinear approaches. The linear mapping algorithm is the fastest one. When the maximum and/or minimum values of a time series are much larger or smaller than the mean/median value of the signal, the majority of *x*_i_ are assigned to only few classes. Thus, we first employ the normal cumulative distribution function (NCDF) to map *x* into $y=\left\{y_{1},y_{2},\ldots ,y_{N}\right\}$ from 0 to 1. Next, we use a linear algorithm to assign each $y_{j}$ to an integer from 1 to *c*. To do so, for each member of the mapped signal, we use $z_{j}^{c}=\mathrm{round}\left(c\cdot y_{j}+0.5\right)$, where $z_{j}^{c}$ shows the *j*^th^ member of the classified time series and rounding involves either increasing or decreasing a number to the next digit. It is worth noting that this step could be done by some other linear and nonlinear mapping techniques.

(2) Each embedding vector $z_{i}^{m,c}$ with embedding dimension *m* and time delay *d* is created according to $z_{i}^{m,c}=\left\{z_{i}^{c},z_{i+d}^{c},\cdots ,z_{i+\left(m-1\right)d}^{c}\right\},i=1,2,\cdots ,N-\left(m-1\right)d$. Each time series $z_{i}^{m,c}$ is mapped to a dispersion pattern $\pi_{v_{0},v_{1},\cdots ,v_{m-1}}$, where $z_{i}^{c}=v_{0}$, $z_{i+d}^{c}=v_{1}$, ⋯, $z_{i+\left(m-1\right)d}^{c}=v_{m-1}$. The number of possible dispersion patterns that can be assigned to each time series $z_{i}^{m,c}$ is equal $c^{m}$, since the signal has *m* members and each member can be one of the integers from 1 to *c*.

(3) For each of $c^{m}$ potential dispersion patterns, relative frequency is obtained as follows:

(9) $$p\left(\pi_{v_{0},v_{1},\cdots ,v_{m-1}}\right)=\frac{Number\{i|i\leq N-\left(m-1\right)d,\;z_{i}^{m,c}\;has\;type\;\pi_{v_{0},v_{1,\cdots ,v_{m-1}}}\}}{N-\left(m-1\right)d}$$

In fact, $p\left(\pi_{v_{0},v_{1},\cdots ,v_{m-1}}\right)$ shows the number of dispersion patterns $\pi_{v_{0},v_{1},\cdots ,v_{m-1}}$ that are assigned to $z_{i}^{m,c}$, divided by the total number of embedding signals with embedding dimension *m*.

(4) Finally, based on the Shannon’s definition of entropy, the DE value with embedding dimension *m*, time delay *d*, and the number of classes *c* is calculated as follows:

(10) $$DE\left(x,m,c,d\right)=-\overset{c^{m}}{\underset{\pi =1}{\sum }}p\left(\pi_{v_{0},v_{1},\cdots ,v_{m-1}}\right)logp\left(\pi_{v_{0},v_{1},\cdots ,v_{m-1}}\right)$$

### 2.5. Wavelet Packet Decomposition

**Figure 3:**
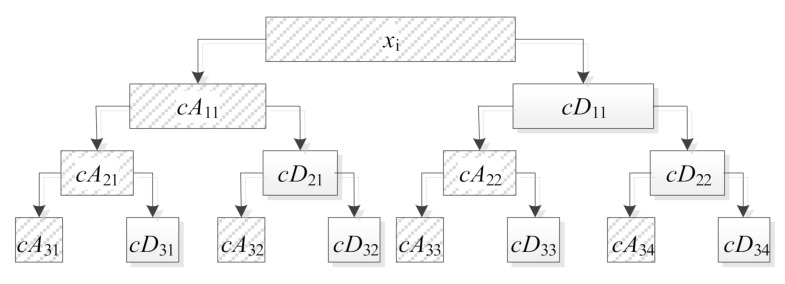
A three-layer decomposition structure diagram.

Features 20 to 27 are the norm of wavelet packet decomposition coefficient reconstruction signal. Wavelet decomposition expands the signal on a series of wavelet basis functions. In engineering applications, because useful signals usually appear as low-frequency parts or some relatively stable signals, interference usually appears as high-frequency signals. Therefore, the signal can be approximated by low-frequency coefficients with a small amount of data and several high-frequency layer coefficients. Figure 3 shows a three-layer decomposition structure diagram, where $cA_{ij}$ and $cD_{ij}$($i=1,2,3\;1\leq j\leq 2^{i-1}$) are the low-frequency and high-frequency decomposition coefficients of the corresponding layer.

Feature extraction based on wavelet decomposition is divided into the following steps:

(1) Wavelet packet decomposition of one-dimensional signal. Select db1 wavelet and determine the level of wavelet decomposition to be 3, and then, perform 3-level wavelet packet decomposition on signal x.

(2) Perform wavelet reconstruction on the decomposed coefficients. According to the low-frequency coefficients of the Nth layer of wavelet decomposition and the high-frequency coefficients of the first to Nth layers, a one-dimensional signal wavelet reconstruction is performed.

(3) Calculate the 2 norms of the reconstructed signal and use them as features F20–F27.

### 2.6. Frequency Domain Feature Set

The frequency domain features include the sum of the spectrum amplitude, the average value of the spectrum, the standard deviation of the spectrum, and the integral of the frequency domain curve, which are represented by F28–F31, respectively.

## 3. Feature Selection Algorithm for Rotating Machinery Noise Diagnosis

The rotating machinery noise diagnosis process generally includes three steps: feature extraction, feature selection (or feature dimension reduction), and state classification.

The traditional feature selection is to separate the data from the classification and map the original features into several features selected by the algorithm by dimension reduction. As shown in Table 2, the characteristics and differences of the commonly used feature filtering algorithms are mainly described, and their methods of processing data have their own focus.

**Figure 4:**
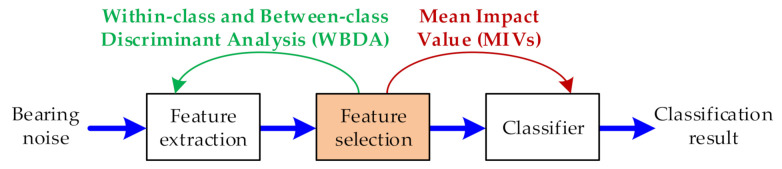
The complementary role of the algorithm in the rolling bearing noise diagnosis process.

Aiming at the problem that the traditional feature selection is usually separated from the learning of prediction model for rotating machinery noise diagnosis, this paper proposes a feature selection algorithm based on network variable selection and within-class and between-class discriminant analysis (WBDA). The proposed algorithm realizes the compromise between the two types of feature selection technique, as shown in Figure 4.

### 3.1. Primary Feature Selection Oriented to the Classification Network—MIVs-SVM

**Figure 5:**
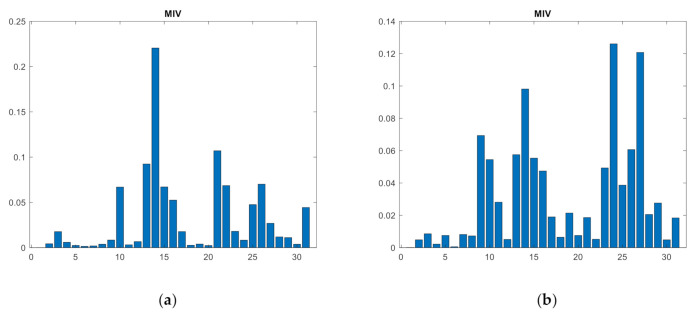

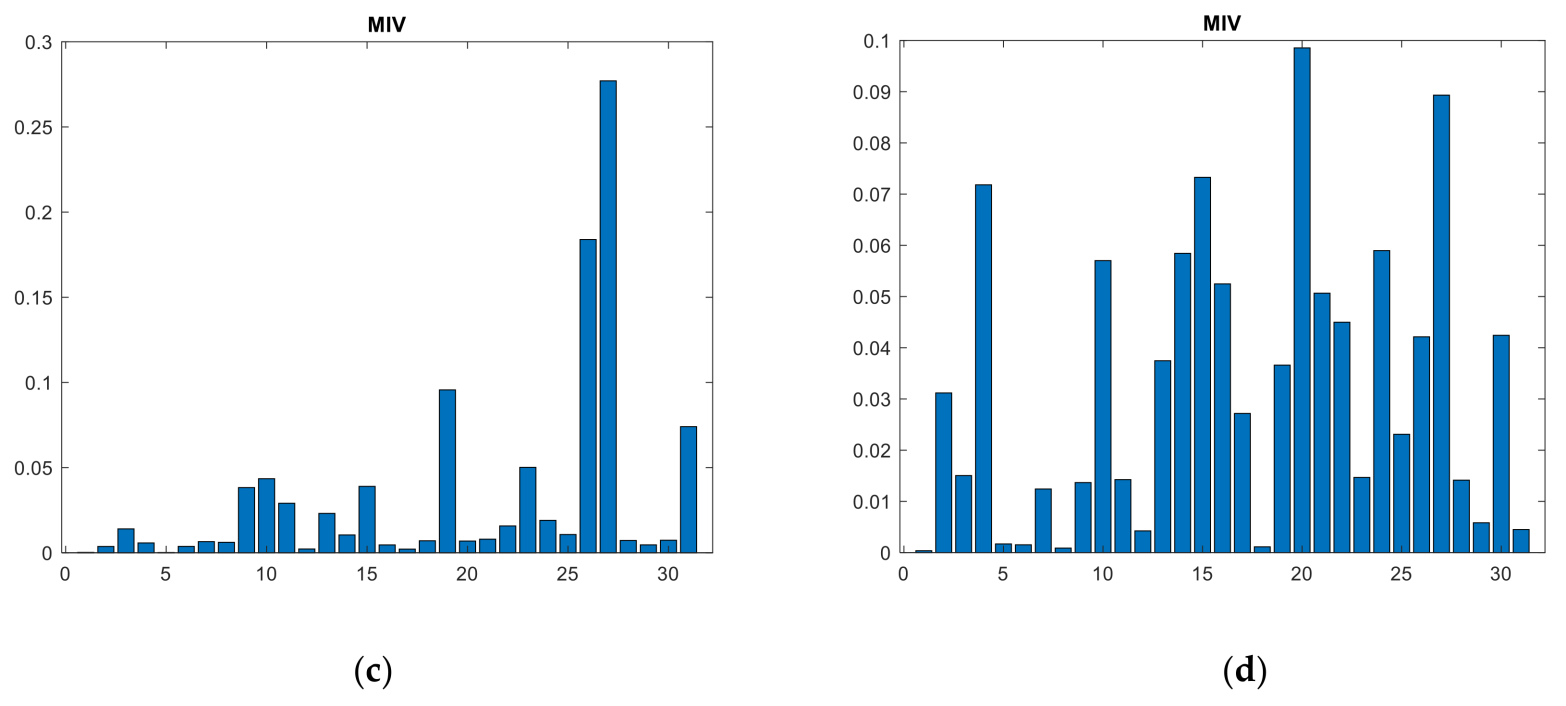
The randomness of mean impact value (MIV) when using neural network to calculate it. (**a**–**d**) show four different results.

The selection of meaningful time-frequency features of noises as SVM input is a key step for status predictions. The MIV is considered as one of the most effective indexes to evaluate the influence of variables on the output of neural network. However, when the neural network is used as classification network to calculate the MIV of the feature variable, the calculated MIVs have great randomness because the parameters of the neural network obtained by each training are not the same. Figure 5 shows the randomness when the neural network is used to calculate MIV. Among them, the abscissa is the characteristic, and the ordinate is the MIV.

**Figure 6:**
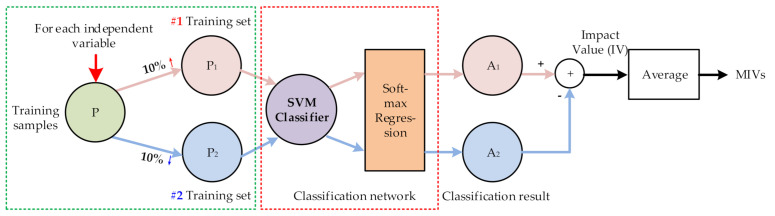
Method for calculating feature MIVs-SVM.

Since SVM is used for fault classification, this algorithm uses SVM network to calculate MIV named MIVs-SVM. Considering that, the final output of SVM is the sample belonging to a class rather than a continuous output value. After the SVM classification hyperplane is obtained by training, the estimation value of posterior probability $P(y_{i}=c|x_{i}),c\in \left\{1,2,\ldots ,N\right\}$ of sample $x_{i}$ belonging to each class *c* is calculated in this paper by Softmax Regression function at first, and then the probability corresponding to the real class of sample $x_{i}$ is selected as the output result. The specific calculation method is shown in Figure 6 and is described as follows:

(a) After the network training, each feature variable in the training sample P was increased and decreased by 10% to obtain training samples P_1_ and P_2_, respectively. P_1_ and P_2_ were input into the established networks, and Softmax Regression function was applied to the output of SVM network. Two new classification results are represented by A_1_ and A_2_.

(b) The difference between A_1_ and A_2_ was obtained and regarded as the impact value (IV) of independent variable variation on the output.

(c) The output MIV of the independent variable on the dependent variable was obtained based on the average IV of all monitoring cases (different fault samples), resulting in the MIV of the specific feature (average of different fault samples).

(d) Repeat steps a–c to obtain the MIV of each feature variable.

(e) The effects of each independent variable on the output were evaluated based on their absolute MIV, and then the effects of the input feature on the results were evaluated, thus achieving variable selection.

Since this modified method directly uses the subsequent classification network SVM to calculate the MIV, it is called MIVs-SVM, abbreviated as MIVs.

### 3.2. Secondary Feature Selection Based on Feature Divisibility—WBDA

The effects of feature variables on the output were sorted based on network feature selection, which reflected the correlation of feature selection algorithms and feature classification algorithms. It provides references for variable selection oriented to the classification network. Nevertheless, to evaluate the divisibility of features, we hope that the features in the same sample are as close as possible, while the features of different samples are as far as possible. To this end, the idea of WBDA was introduced.

The idea of WBDA comes from linear discriminant analysis (LDA). The idea of LDA is very naive: given the set of training samples, try to project the samples onto a straight line, so that the projection points of the same kind of samples are as close as possible, and the projection points of different samples are as far away as possible. LDA is used for feature dimensionality reduction, so it is necessary to construct the optimal linear transformation ***W***. In this case, the purpose of the algorithm is feature selection, so the linear transformation can be omitted. The specific algorithm is described as follows.

For any feature *x_k_*, define within-class divergence

(11) $$J_{w}=\underset{i}{\sum }S_{i}^{2}\;i=1\cdots c$$

where $\;S_{i}^{2}$ is called the divergence of $X_{i}$.

(12) $$S_{i}^{2}=\underset{x\in X_{i}}{\sum }{\left(x-\mu_{i}\right)}^{2}\;i=1\cdots c$$

Define between-class divergence

(13) $$J_{b}=\underset{i\neq j}{\sum }{\left(\mu_{i}-\mu_{j}\right)}^{2}\;i,j=1\cdots c$$

Therefore, the larger $J_{b}$ and the smaller $J_{w}$ are the better. Taking these two points into consideration, the objective function is defined as

(14) $$J=\frac{J_{b}}{J_{w}}=\frac{\sum_{i\neq j}{\left(\mu_{i}-\mu_{j}\right)}^{2}}{\sum_{i}S_{i}^{2}}$$

In order to prevent the phenomenon that the calculated WBDA of the features in the same category are relatively large, so that the selected features do not have the characteristics of diversity, this paper proposes a between-class selection strategy, that is, select the maximum WBDA value of one class each time, then select the maximum value among the remaining classes next time. Once a certain class participates in the selection, it will not participate in the selection of subsequent features until all features in all classes are selected. After that, feature selection will go to the next cycle.

## 4. Classifier and Its Parameter Optimization

Feature classifications were achieved using the SVM. Multi core support vector machine is suitable for complex industrial environment, which requires relatively less hardware resources and has stable classification effect and good generalization performance. Let the training set be $T=\left\{\left(x_{1},y_{1}\right),\left(x_{2},y_{2}\right),\cdots ,\left(x_{m},y_{m}\right)\right\}$, where $x_{i}$ is the *i*-th input data, and $y_{i}\in \left\{-1,1\right\}$ is its corresponding output label. The process of the SVM processing the nonlinear binary classification problem is shown below [30]:

(1) Select the appropriate kernel function $K\left(x_{i},x_{j}\right)=\langle \Phi \left(x_{i}\right)\Phi \left(x_{j}\right)\rangle$ and the appropriate penalty parameter C>0 to construct the following constraint optimization problem:

(15) $$\{\begin{array}{c} \begin{array}{c} \underset{\alpha }{min}\frac{1}{2}\overset{m}{\underset{i=1}{\sum }}\overset{m}{\underset{j=1}{\sum }}\alpha_{i}\alpha_{j}y_{i}y_{j}K\left(x_{i},x_{j}\right)-\overset{m}{\underset{i=1}{\sum }}\alpha_{i}\\ \begin{array}{cc} st. & \overset{m}{\underset{i=1}{\sum }}\alpha_{i}y_{i}=0 \end{array} \end{array}\\ 0\leq \alpha_{i}\leq C,\;i=1,2,\cdots ,m \end{array}$$

where Φ(x) is the mapping function, and $\langle \Phi \left(x_{i}\right)\Phi \left(x_{j}\right)\rangle$ is the inner product of $\Phi \left(x_{i}\right)$ and $\Phi \left(x_{j}\right)$.

(2) Use the sequential minimal optimization (SMO) algorithm to find the optimal solution $\alpha^{*}={\left(\alpha_{1}^{*},\alpha_{2}^{*},\cdots ,\alpha_{m}^{*}\right)}^{T}$ corresponding to the minimum of the above formula.

(3) Calculate the normal vector $w^{*}=\overset{m}{\underset{i=1}{\sum }}\alpha_{i}^{*}y_{i}\phi \left(x_{i}\right)$ of the separated hyperplane, where $w^{*}$ cannot be directly and explicitly evaluated.

(4) Find all of the *S* support vectors $\left(x_{s},y_{s}\right)$ on the maximum interval boundary, and calculate the $b_{s}^{*}=y_{s}-\overset{m}{\underset{i=1}{\sum }}\alpha_{i}y_{i}K\left(x_{i},x_{s}\right)$ corresponding to each support vector. The average of all $b_{s}^{*}$ is the final $b^{*}=\frac{1}{S}\overset{S}{\underset{s=1}{\sum }}b_{s}^{*}$. Thus, the final classification hyperplane is $\overset{m}{\underset{i=1}{\sum }}\alpha_{i}^{*}y_{i}K\left(x,x_{i}\right)+b^{*}=0$, and the classification decision function is

(16) $$f\left(x\right)=\mathrm{sgn}(\overset{m}{\underset{i=1}{\sum }}\alpha_{i}^{*}y_{i}K\left(x,x_{i}\right)+b^{*})$$

The kernel function is equivalent to transforming the original input space into a new feature space through the mapping function and learning the linear support vector machine from the training samples in the new feature space. Learning is implicitly done in the feature space. In practical applications, the choice of kernel function needs to be verified by experiments. The radial basis kernel function is chosen in this paper.

The performance of the SVM classifier is mainly affected by the penalty factor (*C*) and the nuclear parameter (*γ*). The nuclear function mainly reflects the complicity of sample data in high-dimension space, meanwhile, the penalty factor affects the generalization capability of the SVM by tuning the ratio of confidence interval and empiric risk in the feature space. Hence, optimization of SVM performance is usually converted into optimization selection of (*C*, *γ*) by parameters. Conventional optimization algorithms include the PSO algorithm and the GA.

PSO employs the swarm-based global searching strategy and the speed-displacement model and involves no complicated genetic procedures. The unique memory capability of PSO allows dynamic tracking of the current searching situation. Indeed, PSO can be regarded as searching of a swarm consisting of *m* particles $Z=\left\{Z_{1},Z_{2},\ldots ,Z_{m}\right\}$ in an n-dimensional space, and the location of each particle $Z_{i}=\left\{z_{i1},z_{i2},\;\ldots ,z_{in}\right\}$ refers to a solution. The optimized solution of each particle obtained is denoted as $p_{id}$, and the optimized solution in the particle swarm is denoted as $p_{gd}$. The particle speeds are denoted as $V_{i}=\left\{\;v_{i1},v_{i2},\;\ldots ,v_{in}\right\}$ and the renewal rule of *V_i_* in cases of two optimized solutions is as follows [38]:

(17) $$v_{id}\left(t+1\right)=wv_{id}\left(t\right)+\eta_{1}rand()\left(p_{id}-z_{id}\left(t\right)\right)+\eta_{2}rand()\left(p_{gd}-z_{id}\left(t\right)\right)$$

(18) $$z_{id}\left(t+1\right)=z_{id}\left(t\right)+v_{id}\left(t+1\right)$$

where $v_{id}\left(t+1\right)$ refers to the speed of the *i*-th particle at the (*t* + 1)-th iteration in the *d*-th dimension, *w* refers to the weight, $\eta_{1}$ and $\eta_{2}$ refer to acceleration constants, and rand() refers to a random number between 0 and 1.

The GA is a parallel random searching optimization approach that mimics biological evolution [42]. Individuals are selected by selection, cross, and mutation in genetics according to the selected fitness function to retain individuals with good applicability and exclude individuals with poor applicability. In this way, the new generation inherits information from the old generation and outperforms the old generation. This process is repeated until the requirements are satisfied.

Optimizations of network classification parameters by this algorithm classifier were achieved using the two optimization algorithms.

## 5. Network Variable Selection and WBDA Fusion-Oriented Rotating Machinery Noise Diagnosis Algorithm

The network variable selection and WBDA fusion-oriented rotating machinery noise diagnosis algorithm (MIVs-WBDA algorithm) is a feature selection algorithm using network variable selection and WBDA. First, features were selected according to the contributions of independent variables on the classified network, thus achieving classified network oriented variable primary selection. Then, secondary feature selection and dimensionality reduction were achieved according to WBDA, which reflects the divisibility, thus achieving SVM identification. The steps were as follows:

(1) According to the calculated data feature set, samples were randomly divided into training samples, cross-validation reference samples, and testing samples. Cross-validation is a statistical analysis method to validate classifier performance, and experimental results demonstrated that the effectiveness of SVM training based on parameters selected by the cross-validation set was higher than that based on randomly selected parameters. Therefore, the feature MIVs was calculated by cross validation samples.

(2) After excluding *N* features with significant MIVs and features with negligible MIVs, the remaining features were arranged in the order of ascending between-class WBDA. According to the dimensionality after dimensionality reduction (*L*), a new feature vector consisting of the first *L-N* features and *N* features with significant MIVs was generated.

(3) According to the SVM optimization algorithm, the (*C*, *γ*) of the SVM was optimized using the cross-validation set.

(4) We conducted learning based on the training set and tested the identification accuracy of the current SVM.

**Figure 7:**
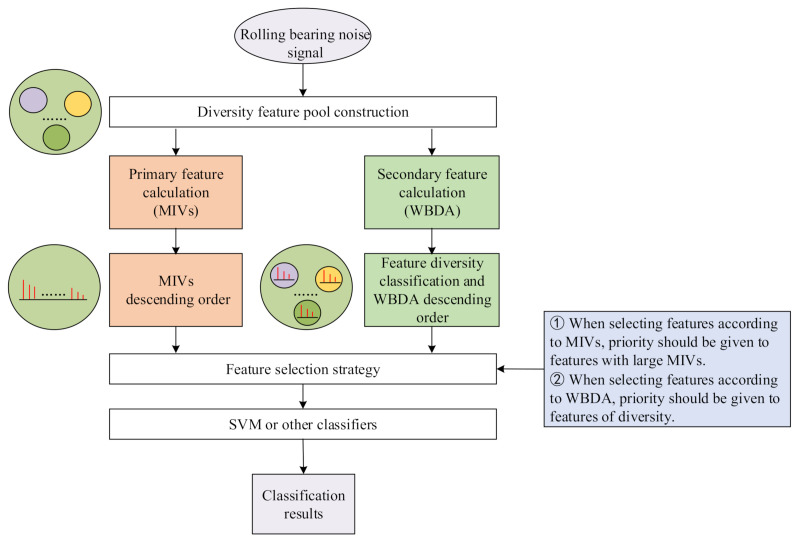
MIVs-WBDA Algorithm.

Figure 7 shows the MIVs-WBDA algorithm flow and the relationship between the two feature selection algorithms and other modules in the algorithm. The result of primary feature selection is controlled by the classifier type, and secondary feature selection is mainly conducted for the residual feature set according to the characteristics of the feature itself. The feature metric chosen for secondary feature selection is the WBDA defined in this paper. Therefore, we produce a feature selection algorithm for network variable selection and WBDA fusion. The superiority of this method is proved in Section 6.

Algorithm 1 summarizes the procedures of the network variable selection and feature entropy fusion oriented bearing noise diagnosis algorithm, including feature extraction and feature classification.
**Algorithm 1.** The MIVs-WBDA Algorithm**Input:** Data set *X*, dimensions after reduction *L* **Output:** Feature set *FS*, classification result *O*, and recognition rate *R* **Step 1:** Calculate the data feature set; randomly assign the training samples, cross-validation samples, and test samples **Step 2:** The MIVs of each feature are calculated using the cross-validation samples, and the most prominent *N* features are selected to form the feature set FS1 **Step 3:** Calculate the between-class WBDA of residual features **Step 4:** Arrange the WBDA from small to large, select the first *L-N* features, and form the feature set FS2. Then form a new special collection FS with FS1. It should be noted that the *L-N* features should be distributed in as many classes as possible. FS = {FS1,FS2} **Step 5:** According to the SVM optimization algorithm, the cross-validation set is used to optimize the selection of support vector machines (*C*, *γ*) **Step 6:** Learn through the training set, and test the SVM output classification result *O* and recognition accuracy *R*

## 6. Results and Discussion

### 6.1. Testing Data

**Figure 8:**
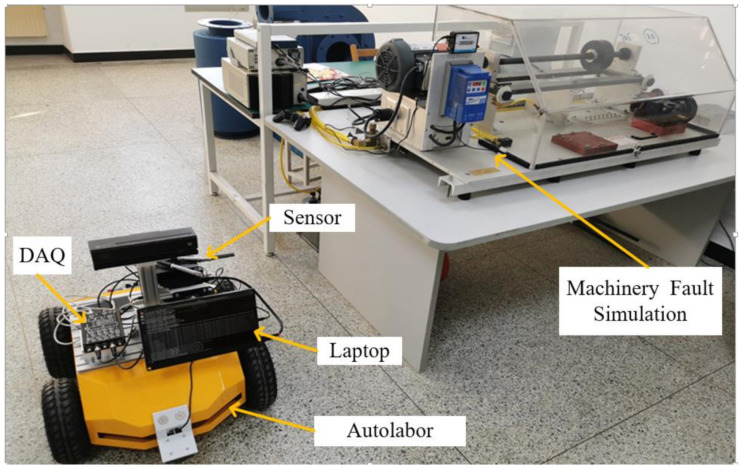
Machinery Fault Simulator^TM.^

**Figure 9:**
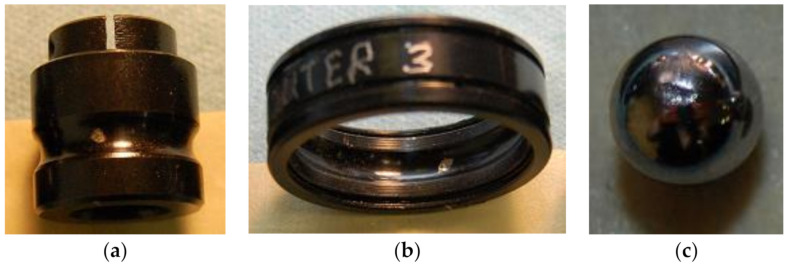
Physical map of three types of faults. (**a**) Inner race fault, (**b**) Outer race fault, (**c**) Ball fault.

**Figure 10:**
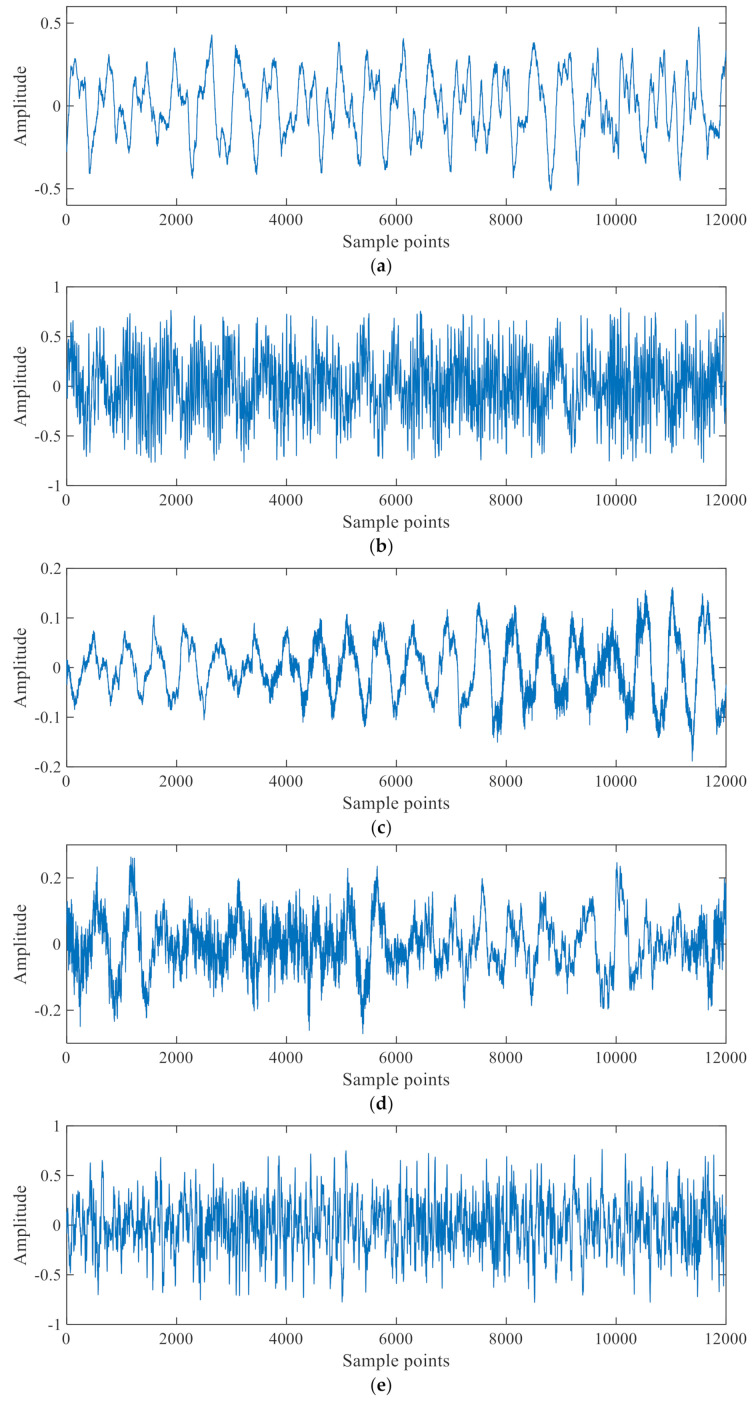
Noise signal of bearing. (**a**) Normal, (**b**) Inner race fault, (**c**) Outer race fault, (**d**) Ball fault, (**e**) Combined fault.

In this experiment, the Machinery Fault Simulator^TM^ MFS-MG2010 was taken as the research object; its mechanical structure is shown in Figure 8, and the specific instrument details are shown in Table 3. The pickup is installed on a moving trolley, so that one device can monitor multiple devices. The fault bearing uses a 1-inch rolling bearing standard fault kit that includes an inner race fault bearing, an outer race fault bearing, a ball fault bearing and a combined fault bearing. Among them, the combined fault bearing is a combination of three fault types: inner race fault, outer race fault, and ball fault. The vibration features can be greatly affected by the fault edge profiles [47]. Figure 9 is a physical map of three types of faults. Figure 10 is Experimental environment and some testing instruments. The fault is a small round hole with a diameter of 2–3 mm and a depth of about 0.5 mm of the testing bearings. The noise signal of five modes (normal, inner race fault, outer race fault, ball fault, and combined fault) at motor speed of 1800 rpm and sampling frequency of 44.1 kHz were obtained and are shown in Figure 10. The signal was obtained through the pickup. The *x*-axis is the number of sampling points, and the *y*-axis is the signal amplitude. Since we only focus on the relative trend of signal amplitude over time and do not pay attention to its actual size, the *y*-axis unit is not marked in the figure; this is common practice [42,48].

### 6.2. Feature Extraction and Classification of Bearing Noise Signal

#### 6.2.1. Feature Extraction

**Figure 11:**
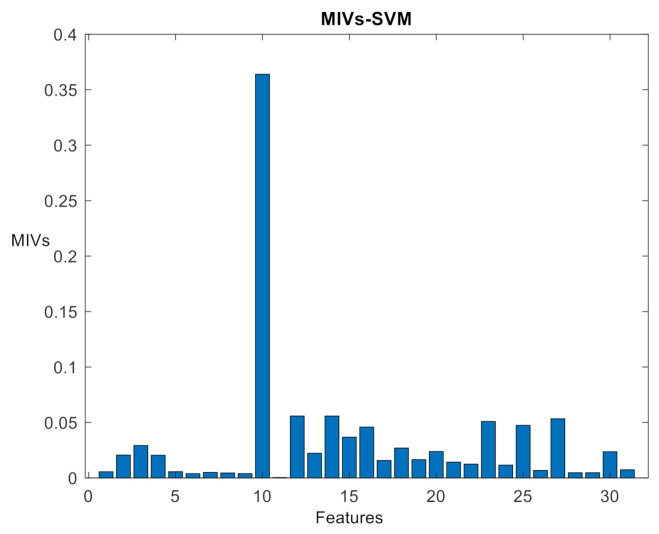
MIVs absolute value distribution of 31 features.

In this study, 720 training sets, 360 cross-validation sets, and 120 testing sets were generated randomly by Matlab. Figure 11 shows the average absolute MIVs and WBDA of samples in the five different clusters with the cross-validation set as the feature set.

**Figure 12:**
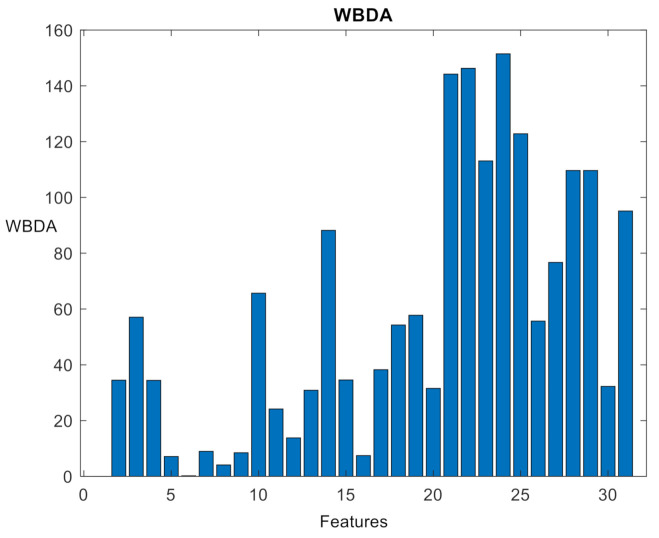
WBDA absolute value distribution of 31 features.

From Figure 11, F10 is significantly different from the other features (and it can be directly selected), but the remaining features have similar MIVs, and thus it is not persuasive to evaluate features with similar MIVs according to the network features. Therefore, the WBDA of the features in the five clusters is calculated using the cross-validation set. The results are shown in Figure 12. According to the between-class selection strategy, based on the WBDA value, the order of feature selection is F24, F29, F14, F10, F19, F18, F22, F28, F3…

**Figure 13:**
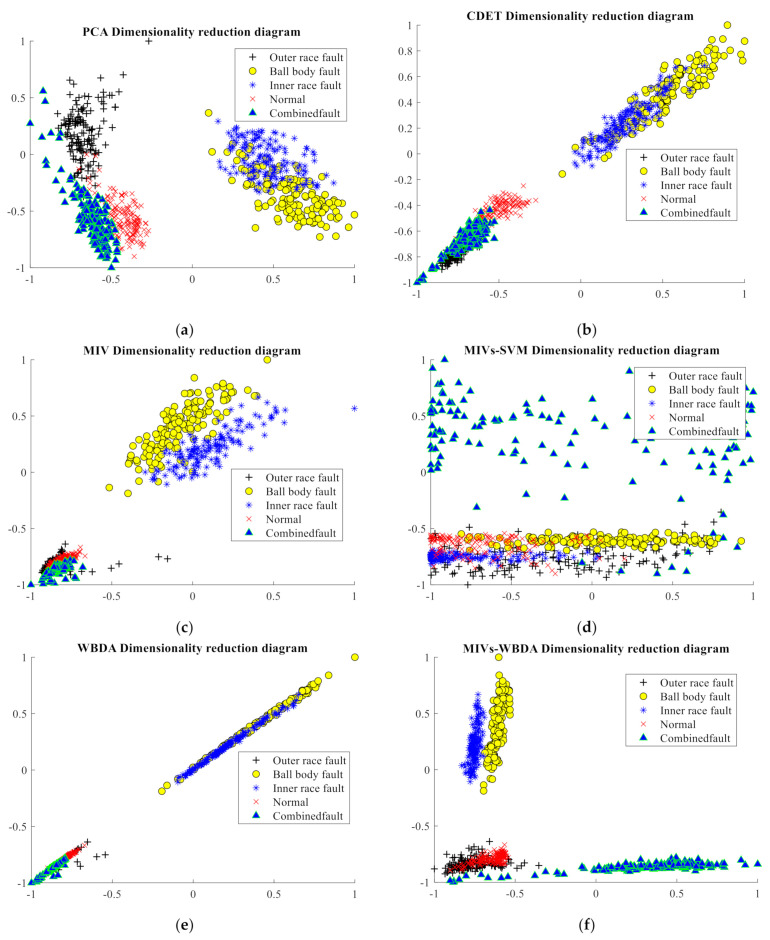
Two-dimensional feature distribution. (**a**) PCA, (**b**) CDET, (**c**) MIV, (**d**) MIVs-SVM, (**e**) WBDA, and (**f**) MIVs-WBDA.

To visualize the dimensionality reduction data, the dimension after reduction was set to be two. We arrange the WBDA in ascending order and select the largest feature. The two-dimensional (2D) feature vector consisting of the largest WBDA (F24), whose corresponding actual features was the norm of three-layer wavelet packet decomposition coefficient $cA_{33}$ and the largest MIVs (margin factor) is the 2D feature vector selected by the MIVs-WBDA algorithm. For easy comparison, Figure 13 shows the 2D feature distributions of the five different clusters for PCA, CDET, MIV, MIVs-SVM, WBDA, and MIVs-WBDA algorithms. Since the five types are nonlinearly separable, it is difficult to see from the figure which feature dimension reduction algorithm works better.

#### 6.2.2. Effects of MIVs-WBDA and Network Optimization Algorithm on the Classification Accuracy of the SVM

**Figure 14:**
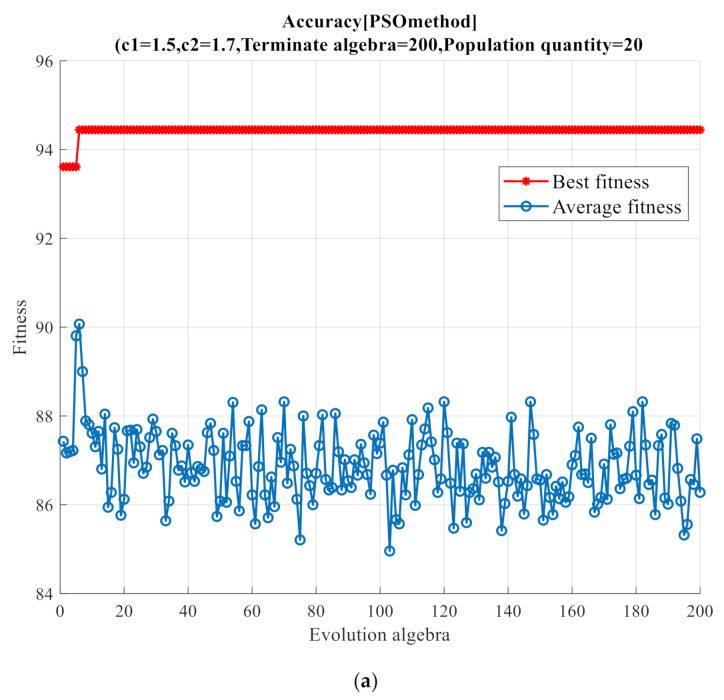

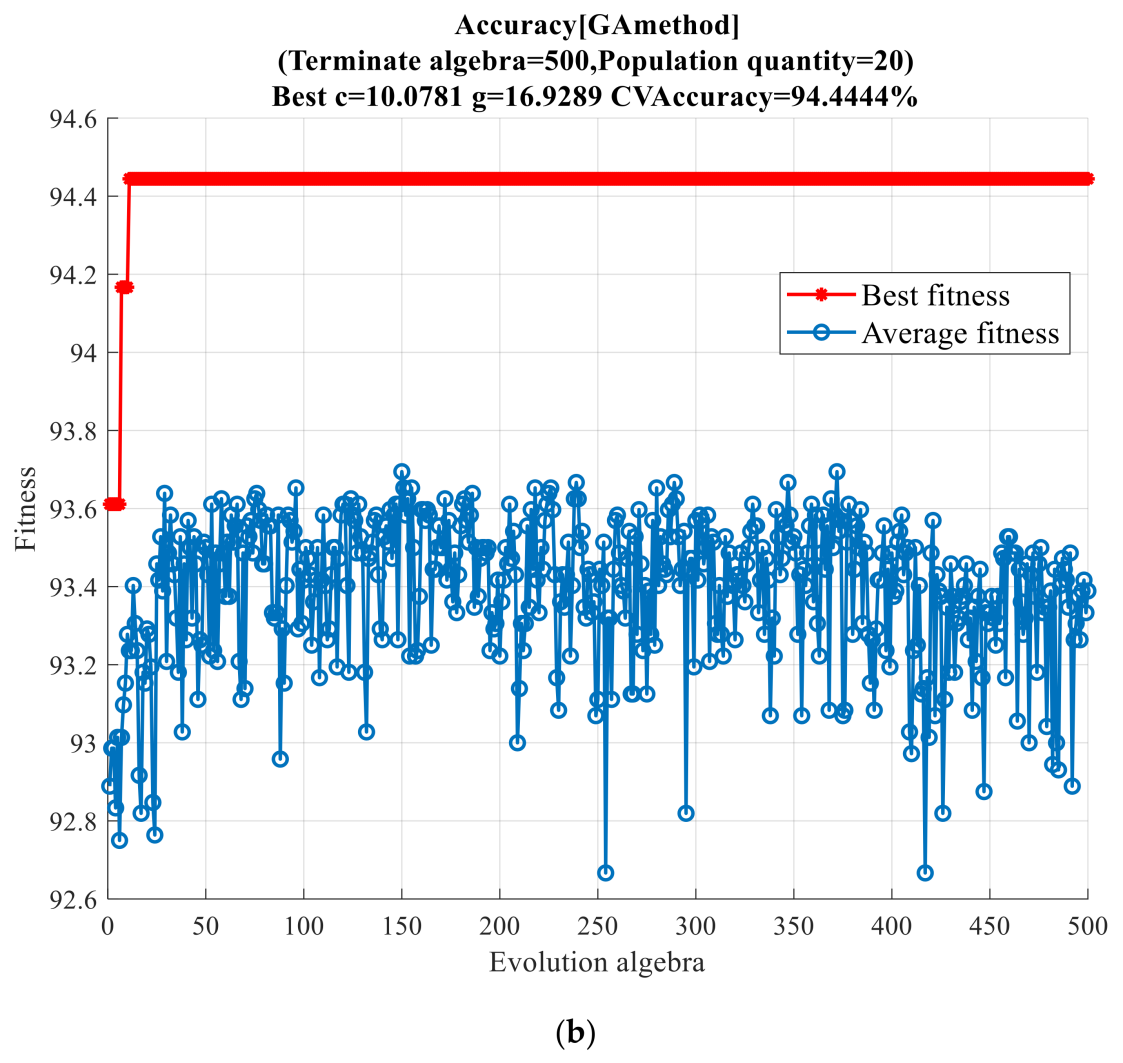
GA algorithm and PSO algorithm running structure. (**a**) PSO and (**b**) GA.

According to the 2D feature vectors obtained by different feature extraction methods, samples were classified into five clusters using PSO, GA optimization, and conventional SVM classifiers. Table 4 summarizes classification accuracies of the different dimensionality reduction and optimization algorithms. In the table, SVM refers to the conventional SVM classifier. It can be seen from the table that the MIVs-WBDA performs better than the other three feature extraction algorithms regardless of whether the optimization algorithm is used. The MIVs-WBDA algorithm exhibited the highest classification accuracy owing to the complementarity of two parts in algorithms. For this example, after using PSO optimization, the MIV-FE algorithm reaches 90.8% classification accuracy. As can be seen from the table, the MIVs-WBDA algorithm has an improvement of classification accuracy of about 3%. Because of reflection, interference, diffraction, and multi-interference sources when the noise signal propagates in the air, the noise diagnosis algorithm is susceptible to the environment, so the classification accuracy is lower than the classification algorithm based on the vibration signal [4]. Figure 14 shows executive procedures of GA and PSO algorithms combined with the MIVs-WBDA method.

**Figure 15:**
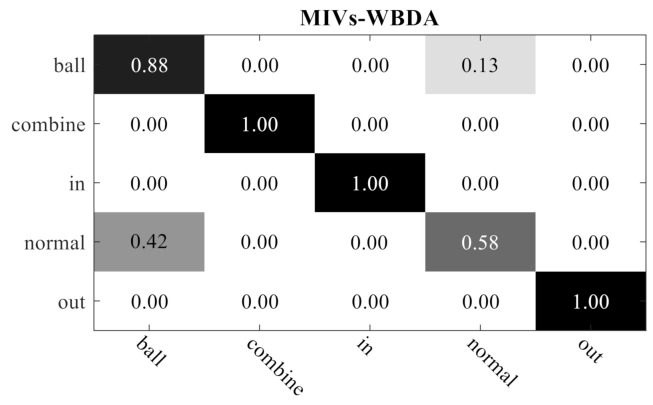
Confusion matrix of the MIVs-WBDA algorithm.

Figure 15 shows the confusion matrix of classification results obtained by the proposed MIVs-WBDA algorithm. As observed, it is difficult to distinguish Normal and Ball when the feature vector dimension is 2. This can also explain why the classification accuracy of the experimental results is just 90%. In fact, the accuracy of noise diagnosis is lower than the diagnosis based on vibration signals. Its typical accuracy is less than 90%.

#### 6.2.3. Effects of Dimensionality Reduction on Performances of Different Algorithm

**Figure 16:**
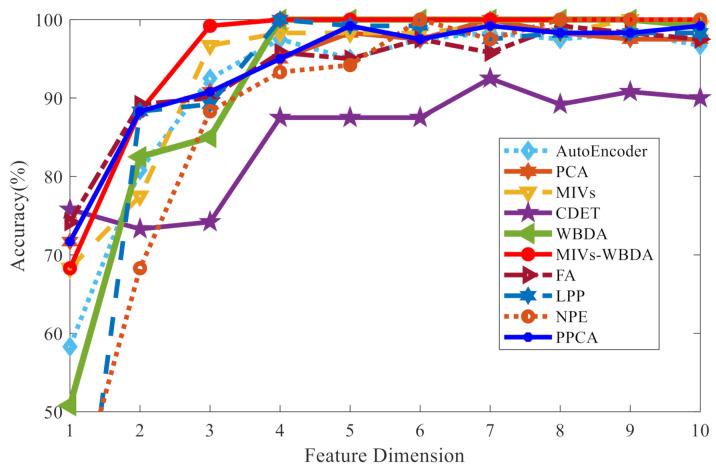
SVM classification accuracy under different dimensions.

Table 5 and Figure 16 illustrate the effects of different feature dimensions on the classification accuracy of the SVM. As observed, when the feature vector dimension is greater than 2, the classification accuracy of MIVs-WBDA is the highest, indicating its excellent feature selection performance. In addition, the function of the classification accuracy and the feature dimension is a concave function.

### 6.3. Algorithm Complexity

**Figure 17:**
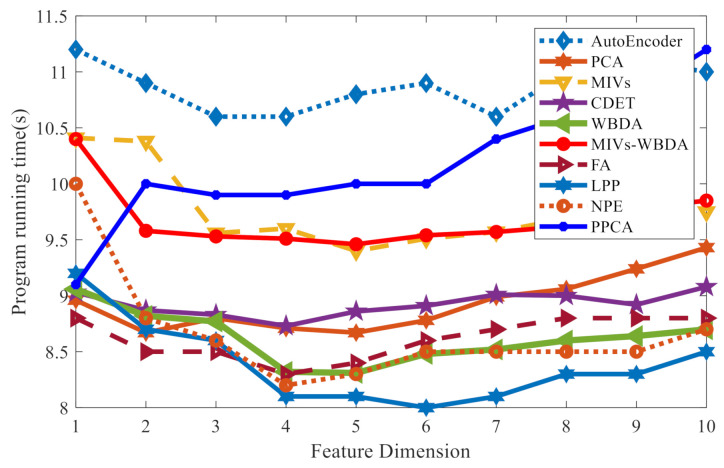
Programming running time under different feature dimension.

The algorithm complexity can be expressed by program runtime. Table 6 presents the testing environment. Table 7 and Figure 17 illustrate the relation between CPU operation time and the feature dimension.

We can analyze the running efficiency of each algorithm through the Table and Figure. It can be seen that the typical running time of most algorithms, including MIVs-WBDA, is less than 10S, which is completely acceptable in practical application. Compared with the traditional methods, the deep learning method has more time overhead, which is more expensive. The above experiments highlight the advantages of MIVs-WBDA through the aspects of operation efficiency and accuracy.

## 7. Conclusions and Future Works

Since redundant information in high-dimension feature vectors may lead to curse of dimensionality and increasing calculation time, this paper proposes an end-to-end feature selection and dimension reduction method (MIVs-WBDA), and compares it to popular PCA, CDET, MIV, FA, LPP, NPE, and PPCA dimensionality reduction methods. Unlike the conventional feature learning algorithm, MIVs-WBDA is a sample feature selection method based on the fusion of network variable selection and WBDA. Moreover, it involves the correlation of feature selection and the classified network and the correlation of the classified network and feature similarity. Hence, the MIVs-WBDA can partially overcome the drawbacks of linear classifications. The classification effect of noise measurement depends on the condition of the environment. Different feature selection may affect the final classification result under different operating environment, and the selection will be different when the environment changes. The common feature selection algorithm only maps the data and does not consider the influence of the data on the classifier. This paper mainly considers the influence of the features on the model classification and integrates the model classification and feature selection organically. The WBDA algorithm considers the generalization performance of the algorithm comprehensively. This paper demonstrates the running time and accuracy of MIVs-WBDA algorithm and several common feature selection algorithms. Finally, the results show that the MIVs WBDA algorithm has a good effect on the basis of considering time and classification accuracy. MIVs-WBDA feature extraction algorithm can screen out several features that are most conducive to classification, which has high application value in practice. MIVs-WBDA can select the most important features and exhibits enhanced classification performance, which realizes the unification of feature representation learning and machine prediction learning. Experiments show that under the condition of reducing to the same dimension, the classification accuracy for rotating machinery status using the MIVs-WBDA method has a 3% classification accuracy improvement under the two feature set construction methods. The typical running time of this classification learning algorithm is less than 10 s, while using deep learning; its running time will be more than a few hours. It should be noted that when the feature dimension is reduced to 1, the classification accuracy of the MIVs-WBDA algorithm is not high. It means that the best feature is not selected at this time, and we can consider how to introduce other strategies to solve the accuracy problem when the dimension is 1. In the later stage, the idea of feature extraction can be combined to achieve the improvement of classification performance in low dimensions. Of course, in practical applications, the dimension of the feature vector will not only take one dimension. Therefore, it will not affect the use of this algorithm. The idea of constructing diversity feature pool, end-to-end feature selection and prediction model learning can also be applied to other similar application scenarios.

## Figures and Tables

**Table 1:** Time domain features.

Feature	Name	Definition
F1	Mean	$F_{1}=\frac{1}{N}\overset{N}{\underset{i=1}{\sum }}x_{i}$
F2	Standard deviation (Std)	$F_{2}=\sqrt{\frac{1}{N}\overset{N}{\underset{i=1}{\sum }}{\left(x_{i}-\mu \right)}^{2}}$
F3	Peak to peak	$F_{3}=ximin_{imax}$
F4	Root Mean Square (RMS)	$F_{4}=\sqrt{\frac{1}{N}\overset{N}{\underset{i=1}{\sum }}x_{i}^{2}}$
F5	Kurtosis	$F_{5}=\frac{1}{N\sigma^{4}}\overset{N}{\underset{i=1}{\sum }}{\left(x_{i}-\bar{x}\right)}^{4}$
F6	Skewness	$F_{6}=\frac{1}{N\sigma^{3}}\overset{N}{\underset{i=1}{\sum }}{\left(x_{i}-\bar{x}\right)}^{3}$
F7	Crest Factor	$F_{7}=\frac{max|x_{i}|}{RMS}$
F8	Shape Factor	$F_{8}=\frac{RMS}{\frac{1}{N}\sum_{i=1}^{N}\left|x_{i}\right|}$
F9	Impulse Factor	$F_{9}=\frac{max|x_{i}|}{\frac{1}{N}\sum_{i=1}^{N}\left|x_{i}\right|}$
F10	Margin Factor	$F_{10}=\frac{max|x_{i}|}{{\left(\frac{1}{N}\sum_{i=1}^{N}\sqrt{\left|x_{i}\right|}\right)}^{2}}$
F11	Number of zero crossing points	$F_{11}=\frac{1}{2}\overset{N-1}{\underset{i=1}{\sum }}\left|\mathrm{sgn}\left(x_{i+1}\right)-\mathrm{sgn}\left(x_{i}\right)\right|$

**Table 2:** Feature selection algorithm.

Feature Selection	Characteristics
PCA	PCA transform the original data into linearity independence data via linear transformation.
Probability PCA (PPCA)	PCA does not consider the probability distribution of data, PPCA makes a probability interpretation of PCA, and extends the PCA algorithm.
Autoencoder	Autoencoder is a method of deep learning, which maps data to low dimension feature space by unsupervised method.
CDET	CDET measures the importance of each feature by examining the performance of samples within and between classes.
Locality Preserving Projections (LPP)	LPP constructs the distant and distant relationship between the sample pairs in the space, and maintains this relationship in the projection, so as to reduce the dimension and retain the local neighborhood structure of the samples in the space.
Neighborhood Preserving Embedding (NPE)	NPE is obtained by approximately linear representation of neighborhood, and the best effect is achieved by minimizing the reconstruction error.
MIV	MIV is an evaluation standard, which highlights the impact of the original signal changes on the classification error, and then selects the features.
Factor Analysis (FA)	FA is a statistical analysis method in which a few factors are used to describe the relationship between many indicators or factors and a few factors are used to reflect most of the information of the original data.

**Table 3:** Experimental environment and testing instruments.

Machinery Fault Simulation	DAQ	Sensors	Auto Labor	Laptop
SpectraQuest—MFS RDS	MCC WebDAQ504	SKC NP21	Autolabor Pro1	Autolabor PC

**Table 4:** SVM classification accuracy of noise signals under different dimensionality reduction and optimization algorithms.

Optimization Method	PSO-SVM	GA-SVM	SVM
**PCA**	87.5%	84.2%	88.3%
**CDET**	74.2%	74.2%	73.3%
**MIVs**	77.5%	80%	77.5%
**WBDA**	80.8%	80%	82.5%
**MIVs-WBDA**	90.8%	90%	88.3%

**Table 5:** Classification accuracy of different algorithms in different feature dimensions.

Method	1	2	3	4	5	6	7	8	9	10
Autoencoder	58.3	80.8	92.5	97.5	86.7	97.5	98.3	97.5	98.3	96.7
PCA	71.7	88.3	90.8	95.0	98.3	97.5	100.0	98.3	97.5	97.5
MIVs	68.3	77.5	96.7	98.3	98.3	98.3	99.2	98.3	100.0	99.2
CDET	75.8	73.3	74.2	87.5	87.5	87.5	92.5	89.2	90.8	90.0
WBDA	50.8	82.5	85.0	100.0	100.0	100.0	100.0	100.0	100.0	99.2
MIVs-WBDA	68.3	88.3	99.2	100.0	100.0	100.0	100.0	100.0	100.0	100.0
FA	74.2	89.2	90.0	95.8	95.0	97.5	95.8	99.2	98.3	97.5
LPP	20.0	88.3	89.2	100.0	99.2	99.2	97.5	98.3	98.3	98.3
NPE	35.8	68.3	88.3	93.3	94.2	100.0	97.5	100.0	100.0	100.0
PPCA	71.67	88.33	90.8	95.0	99.2	97.5	99.2	98.3	98.3	99.2

**Table 6:** The testing environment.

CPU	Memory	Hard disk	Operating system	Simulation tool
Intel(R) Core™ i9-9900K @3.6 GHz	32 GB	512 GB	Win10 Profession	Matlab R2018a

**Table 7:** Running time of algorithm.

Method	1	2	3	4	5	6	7	8	9	10
Autoencoder	11.17	10.87	10.60	10.63	10.79	10.85	10.64	10.99	11.17	10.87
PCA	8.96	8.67	8.80	8.71	8.67	8.78	8.99	9.06	9.24	9.43
MIVs	10.41	10.38	9.56	9.60	9.40	9.51	9.57	9.69	9.75	9.75
CDET	9.03	8.87	8.83	8.73	8.86	8.91	9.01	9.00	8.92	9.08
WBDA	9.06	8.82	8.77	8.32	8.31	8.48	8.52	8.60	8.64	8.70
MIVs-WBDA	10.40	9.58	9.53	9.51	9.46	9.54	9.57	9.62	9.78	9.85
FA	8.82	8.54	8.49	8.33	8.37	8.57	8.73	8.77	8.79	8.75
LPP	9.19	8.66	8.61	8.08	8.07	8.03	8.13	8.29	8.31	8.51
NPE	10.03	8.84	8.56	8.24	8.33	8.49	8.52	8.46	8.55	8.71
PPCA	9.08	10.00	9.94	9.92	9.99	10.03	10.42	10.56	10.82	11.18

## Data Availability

The datasets in this paper can be obtained from the following link: https://github.com/waitf10/MIVs-WBDA/tree/main.

## References

[1] William P.E., Hoffman M.W. (2011). Identification of bearing faults using time domain zero-crossings. Mech. Syst. Signal Process..

[2] Zhang X., Liang Y., Zhou J. (2015). A novel bearing fault diagnosis model integrated permutation entropy, ensemble empirical mode decomposition and optimized SVM. Measurement.

[3] Benko U., Petrovcic J., Juricic D., Tavčar J., Rejec J., Stefanovska A. (2004). Fault diagnosis of a vacuum cleaner motor by means of sound analysis. J. Sound Vib..

[4] He Z., Chen J., Wang T., Chu F. (2010). Theories and applications of machinery fault diagnostics. High. Educ. Press.

[5] Vaidya K.S., Parker R.G. (2021). Space-fixed formulation for the vibration of rotating, prestressed, axisymmetric bodies and shells. J. Sound Vib..

[6] Liu R., Yang B., Zio E., Chen X. (2018). Artificial intelligence for fault diagnosis of rotating machinery: A review. Mech. Syst. Signal Process..

[7] Bayar N., Darmoul S., Hajri-Gabouj S., Pierreval H. (2015). Fault detection, diagnosis and recovery using Artificial Immune Systems: A review. Eng. Appl. Artif. Intell..

[8] Li R., Sopon P., He D. (2012). Fault features extraction for bearing prognostics. J. Intell. Manuf..

[9] Lu S., Wang X., He Q., Liu F., Liu Y. (2016). Fault diagnosis of motor bearing with speed fluctuation via angular resampling of transient sound signals. J. Sound Vib..

[10] Liu J., Xu Z., Zhou L., Yu W., Shao Y. (2019). A statistical feature investigation of the spalling propagation assessment for a ball bearing. Mech. Mach. Theory.

[11] Ding X., Li Q., Lin L., He Q., Shao Y. (2019). Fast time-frequency manifold learning and its reconstruction for transient feature extraction in rotating machinery fault diagnosis. Measurement.

[12] Mohanty S., Gupta K.K., Raju K.S. (2018). Hurst based vibro-acoustic feature extraction of bearing using EMD and VMD. Measurement.

[13] Lv Y., Pan B., Yi C., Ma Y. (2019). A Novel Fault Feature Recognition Method for Time-Varying Signals and Its Application to Planetary Gearbox Fault Diagnosis under Variable Speed Conditions. Sensors.

[14] Zhao H., Zhang J., Jiang Z., Wei D., Zhang X., Mao Z. (2019). A New Fault Diagnosis Method for a Diesel Engine Based on an Optimized Vibration Mel Frequency under Multiple Operation Conditions. Sensors.

[15] Dong Z., Zheng J., Huang S., Pan H., Liu Q. (2019). Time-Shift Multi-scale Weighted Permutation Entropy and GWO-SVM Based Fault Diagnosis Approach for Rolling Bearing. Entropy.

[16] Yan X., Jia M., Zhao Z. (2018). A novel intelligent detection method for rolling bearing based on IVMD and instantaneous energy distribution-permutation entropy. Measurement.

[17] Yan X., Jia M. (2019). Intelligent fault diagnosis of rotating machinery using improved multiscale dispersion entropy and mRMR feature selection. Knowl. Based Syst..

[18] Zheng J., Dong Z., Pan H., Ni Q., Liu T., Zhang J. (2019). Composite multi-scale weighted permutation entropy and extreme learning machine based intelligent fault diagnosis for rolling bearing. Measurement.

[19] Rodriguez N., Barba L., Alvarez P., Cabrera-Guerrero G. (2019). Stationary Wavelet-Fourier Entropy and Kernel Extreme Learning for Bearing Multi-Fault Diagnosis. Entropy.

[20] Luo S., Yang W., Tang H. (2020). A novel feature selection method to boost variable predictive model–based class discrimination performance and its application to intelligent multi-fault diagnosis. Meas. Control.

[21] Dai J., Tang J., Shao F., Huang S., Wang Y. (2019). Fault Diagnosis of Rolling Bearing Based on Multiscale Intrinsic Mode Function Permutation Entropy and a Stacked Sparse Denoising Autoencoder. Appl. Sci..

[22] Gu Y.K., Zhou X.Q., Yu D.P., Shen Y.J. (2018). Fault diagnosis method of rolling bearing using principal component analysis and support vector machine. J. Mech. Sci. Technol..

[23] Chen B., Li H., Yu H., Wang Y. (2017). A Hybrid Domain Degradation Feature Extraction Method for Motor Bearing Based on Distance Evaluation Technique. Int. J. Rotating Mach..

[24] Lei Y., He Z., Zi Y., Chen X. (2008). New clustering algorithm-based fault diagnosis using compensation distance evaluation technique. Mech. Syst. Signal Process..

[25] Kuncan M., Kaplan K., Minaz M.R., Kaya Y., Ertunc H.M. (2020). A novel feature extraction method for bearing fault classification with one-dimensional ternary pattern. ISA Trans..

[26] Longbo X.U., Wei W.A.N.G., Zhang T., Li Y.A.N.G., Shaoyong W.A.N.G. (2017). Ultra-short-term Wind Power Prediction Based on Neural Network and Mean Impact Value. Autom. Electr. Power Syst..

[27] Madhusudana C.K., Kumar H., Narendranath S. (2018). Fault Diagnosis of Face Milling Tool using Decision Tree and Sound Signal. Mater. Today Proc..

[28] Li Z., Liu Z., Ding G. (2019). Feature selection algorithm based on XGBoost. J. Commun..

[29] Shao H., Jiang H., Zhao H., Wang F. (2017). A novel deep autoencoder features a learning method for rotating machinery fault diagnosis. Mech. Syst. Signal Process..

[30] Duan Z., Yuan X., Xiong Y. (2016). Fault Diagnosis of Gearbox based on the Optimized BP Neural Networks by Improved Particle Swarm Algorithm. Adv. Comput. Sci. Res..

[31] Guo S., Yang T., Gao W., Zhang C. (2018). A Novel Fault Diagnosis Method for Rotating Machinery Based on a Convolutional Neural Network. Sensors.

[32] Yao C., Lai B., Chen D., Sun F., Lyu S. (2017). Fault Diagnosis Method Based on MED-VMD and Optimized SVM for Rolling Bearings. China Mech. Eng..

[33] Jiang Q., Chang F. (2019). A novel rolling-element bearing faults classification method combines lower-order moment spectra and support vector machine. J. Mech. Sci. Technol..

[34] Han T., Jiang D., Zhao Q., Wang L., Yin K. (2018). Comparison of random forest, artificial neural networks and support vector machine for intelligent diagnosis of rotating machinery. Trans. Inst. Meas. Control.

[35] Hu Q., He Z., Zhang Z., Zi Y. (2007). Fault diagnosis of rotating machinery based on improved wavelet package transform and SVMs ensemble. Mech. Syst. Signal Process..

[36] Liu Y.Q., Yang S.P., Liao Y.Y., Wang C. (2016). The adaptive resonant demodulation method and its application in failure diagnosis of rolling bearing early faults. J. Vib. Eng..

[37] Zhu X., Xiong J. (2018). Fault Diagnosis of Rotation Machinery based on Support Vector Machine Optimized by Quantum Genetic Algorithm. IEEE Access.

[38] Han D., Zhao N., Shi P. (2019). Gear fault feature extraction and diagnosis method under different load excitation based on EMD, PSO-SVM and fractal box dimension. J. Mech. Sci. Technol..

[39] Song W., Liu S., Liu Q. (2009). Business process mining based on simulated annealing. Acta Electron. Sin..

[40] Zhang H., Tao R., Li Z.Y., Cai Z.H. (2009). A research and application of feature selection based on KNN and tabu search algorithms in the intrusion detection. Acta Electron. Sin..

[41] Zan T., Wang H., Wang M., Liu Z., Gao X. (2019). Application of Multi-Dimension Input Convolutional Neural Network in Fault Diagnosis of Rolling Bearings. Appl. Sci..

[42] Zhou S., Qian S., Chang W., Xiao Y., Cheng Y. (2018). A Novel Bearing Multi-Fault Diagnosis Approach Based on Weighted Permutation Entropy and an Improved SVM Ensemble Classifier. Sensors.

[43] Khan M.A., Karim M., Kim Y. (2018). A Two-Stage Big Data Analytics Framework with Real World Applications Using Spark Machine Learning and Long Short-Term Memory Network. Symmetry.

[44] Ali J.B., Fnaiech N., Saidi L., Chebel-Morello B., Fnaiech F. (2015). Application of empirical mode decomposition and artificial neural network for automatic bearing fault diagnosis based on vibration signals. Appl. Acoust..

[45] Sun Y., Li S., Wang X. (2021). Bearing fault diagnosis based on EMD and improved Chebyshev distance in SDP image. Measurement.

[46] Rostaghi M., Azami H. (2016). Dispersion Entropy: A Measure for Time-Series Analysis. IEEE Signal Process. Lett..

[47] Liu J., Shao Y. (2018). An improved analytical model for a lubricated roller bearing including a localized defect with different edge shapes. J. Vib. Control.

[48] Tzanetakis G., Cook P. (2002). Music genre classification of audio signals. IEEE Trans. Speech Audio Process.

